# Role of Long Noncoding RNA Dio3os in Glycolipid Metabolism

**DOI:** 10.2174/0113892029345945241125064704

**Published:** 2025-01-07

**Authors:** Xinchen Wang, Shiyun Zeng, Yuting Liu, Yulan Shi, Fenghua Qu, Li Li, Qirui Zhang, Ding Yuan, Chengfu Yuan

**Affiliations:** 1 Third-grade Pharmacological Laboratory on Traditional Chinese Medicine, State Administration of Traditional Chinese Medicine, China Three Gorges University, China;; 2 Hubei Key Laboratory of Tumor Microenvironment and Immunotherapy, China Three Gorges University, China;; 3 College of Medical and Health Sciences, China Three Gorges University, Yichang 443002, China;; 4 College of Basic Medical Science, China Three Gorges University, Yichang 443002, China

**Keywords:** Glycolipid metabolism, lncRNA, Dio3os, ceRNA network, generational obesity, diabetic peripheral neuropathy

## Abstract

**Introduction:**

Recent investigations have underscored the importance of long non-coding RNAs (lncRNAs), which exhibit more specific expression in tissues and cells than mRNA and are involved in gene regulation during development, pathology, and other processes through various mechanisms. Despite the predominant focus on the role of lncRNA Dio3os in cancer research, there has been relatively limited exploration of its potential involvement in glycolipid metabolism. Therefore, this study aims to consolidate existing knowledge on the function of Dio3os in glycolipid metabolism and calls for a broader investigation into its physiological roles.

**Methods:**

This review synthesizes available literature to detail the gene characteristics of lncRNA Dio3os and its expression patterns. It also compiles recent insights and mechanisms pertaining to Dio3os's involvement in glycolipid metabolism, particularly its participation in the ceRNA regulatory network.

**Results:**

Recent studies demonstrate that lncRNA Dio3os regulates glycolysis in cancer cells and impacts obesity, potentially serving as an indicator for diabetic peripheral neuropathy. Furthermore, its diminished expression has been noted in atherosclerotic plaques.

**Conclusion:**

lncRNA Dio3os exerts a significant regulatory influence on glycolipid metabolism, with variations in its expression levels potentially affecting disease presentations. Further investigations are warranted to elucidate the precise relationship between lncRNA Dio3os and its associated pathologies.

## 1 INTRODUCTION

With advancements in scientific research and an intensified focus on the genetic level, it has become clear that the regulation of gene expression is a domain replete with discoveries and challenges. While the core principle of molecular biology states that DNA functions as a template for transcription and mRNA acts as a template for protein translation, recent research has revealed numerous deviations from this dogma [1].

A growing body of research has acknowledged the significance of non-coding RNAs. Multiple studies have demonstrated that nearly three-quarters of the human genome undergo transcription, yet only a small fraction, approximately 2%, of these transcripts actually encode proteins [2, 3]. The vast majority, estimated to range from 2,000 to 100,000, are transcribed into long non-coding RNAs (lncRNAs) [2, 3]. For example, lncRNAs play a crucial role in shaping chromatin structure, as evidenced by their ability to influence local DNA methylation and histone modifications on nucleosomes, in addition to participating in R-loop formation [3]. At the transcriptional level, lncRNAs can interact with promoters or enhancers, directly influencing transcription by either facilitating or inhibiting the activity of transcriptional proteins [3, 4]. Furthermore, at the protein level, the interactions between lncRNAs and proteins are critical, impacting cellular functions by modifying mRNA stability and localization, as well as regulating the translational and post-translational modifications of proteins [3, 4]. In conclusion, the importance of lncRNAs in regulating gene expression is increasingly recognized.

Among these, Dio3os, a long non-coding RNA, was initially identified as the upstream antisense strand of Dio3, located at the chromosomal locus 14q32.31. As an allele of D io3, Dio3os reflects the developmental and circadian expression patterns of Dio3 [4, 5]. Positioned in close proximity and antisense orientation to Dio3, Dio3os partially overlaps with the Dio3 promoter region [5, 6]. These transcripts exhibit a sense/antisense relationship and belong to the DLK1/DIO3 imprinting cluster at its distal end. Genomic imprinting, characterized by the selective expression of genes based on parental origin, is modulated by antisense imprinted lncRNAs [7]. Such lncRNAs can influence gene expression within the imprinted region either by directly overlapping with a gene or its promoter or by inhibiting the expression of forward-translated alleles from the parent chromosome [7-11]. Within the Dlk1-Dio3 domain, Dio3os acts as an “antisense” maternal allele. Research by Hai-Qiang Xie *et al.* demonstrated that Dio3os mirrors the expression pattern of Dio3 in mice, and changes in Dio3os expression levels correspondingly affect Dio3 expression [24]. This research suggests that the imprinted lncRNA Dio3os may play a regulatory role in the expression of certain genes located within the Dlk1-Dio3 imprinting domain [24].

The expression level of imprinted genes is pivotal for elucidating genetics, growth, and metabolism [12]. Notably, H19 has been linked to a diverse range of metabolic disorders, encompassing cancer, atherosclerosis, non-alcoholic fatty liver disease, and diabetes [13]. Within the Dlk1-Dio3 region, the Dio3 gene holds a crucial position, serving as a key protector of bodily tissues against the damaging impacts of excessive thyroid hormones during the developmental phase [14]. Additionally, the aberrant expression of the lncRNA KCNQ1OT1 is significantly linked to a spectrum of human diseases [15]. Dio3os, another imprinted gene, is involved in the progression of several cancers, including hepatocellular carcinoma [16], non-small cell lung cancer [17], thyroid cancer [18], pancreatic cancer [19], and breast cancer [20]. Recent research by Chen YT *et al.* has highlighted Dio3os's contribution to brown adipose tissue development and its impact on generational obesity [21]. Studies by Serdal Arslan *et al.* have observed a reduction in Dio3os expression in atherosclerosis cases [22], while other research associates Dio3os with the development of diabetic peripheral neuropathy [23]. While the involvement of Dio3os in cancer is well-documented, its role in glycolipid metabolism is less understood. This paper seeks to synthesize current knowledge on the relationship between lncRNA Dio3os and glycolipid metabolic diseases, focusing on its regulatory effects on glucose and lipid metabolism (Table **1**).

## 2 CHARACTERIZATION OF LNCRNA DIO3OS

Dio3os was identified through an in-depth analysis of the Dio3 locus structure, located downstream of the imprinted Dlk1-Dio3 genomic region on mouse chromosome 12F1 and human chromosome 14q32 [5]. In both species, Dio3os overlaps with a G+C-rich segment that constitutes 80% of its sequence, positioned 1.5 kb upstream of Dio3 [7]. Sequencing revealed that mouse Dio3os spans 2312kb, is poly(A) tailed (GenBank accession number MF399212), comprises 15 open reading frames, lacks protein-coding sequences, and exhibits low conservation [24]. Humans possess six Dio3os transcripts, each featuring a conserved exon-intron structure [4, 25]. The Dio3os promoter region contains CpG islands, crucial for genomic regulation, where CpG456 methylation leads to Dio3os silencing [17]. Additionally, RNA interference between Dio3os and Dio3 can suppress Dio3os transcription [26, 27]. In endometriosis cases, elevated ALKBH activity, an m6A demethylase, enhances RNA stability and Dio3os expression by altering its m6A methylation status [28]. Recent research has revealed a pivotal function of lncRNAs in epigenetically regulating gene expression. Specifically, EZH2, a chromatin modifier within the Polycomb Repression Complex 2 (PRC2), enhances transcriptional repression by methylating lysine 27 of histone H3 (H3K27) [29]. This interaction between lncRNAs and EZH2 highlights their importance in regulating gene expression patterns. Dio3os has an inverse correlation with EZH2 in prostate cancer (PRAD), breast cancer (BRCA), and glioblastoma multiforme (GBM) across various cell lines, underscoring the function of EZH2 in repressing Dio3os transcription [30].

Genomic imprinting, a mammalian-specific epigenetic phenomenon, leads to parent-of-origin-specific expression patterns essential for growth, brain function, nutritional metabolism, and behavioral development [31, 32]. Recent microarray analyses have shown that imprinted genes on human chromosome 14 display parent-specific and tissue-specific expression patterns [32]. A particular subset of human cell lines displayed co-expression of D3 and Dio3os, while a distinct set of human neuroblastoma cell lines showed co-expression of D3, Dio3os, and Dlk1 [33]. Paternal imprinting of Dio3 during fetal development occurs, which suggests a potential regulatory role for Dio3os in maintaining the expression of paternally inherited Dio3 alleles [34]. Research on Dio3os in mice demonstrates its regulatory role on Dio3 and Dlk1 expression, associating it with the Dlk1-Dio3 imprinting locus [11]. Beyond gene-specific factors, distal co-regulatory elements are crucial in gene expression modulation. In rats, Dio3os transcripts show a distinct pattern of imprinted expression in a specific brain region, coordinating their expression with Dio3 on the same allele [8]. Hernandez *et al.* observed significant upregulation of Dio3os in rat brown adipose tissue [56]. In mice, biallelic expression, instead of imprinting, occurred in various developmental and adult tissues [11, 31-36].

Additionally, Dio3os is highly expressed in cattle liver, uterus, lungs, ovaries, and kidneys but shows low expression in the heart and muscle, indicating monoallelic expression in multiple tissues and suggesting imprinting-mediated regulation in cattle [7]. Martinez and his team discovered a preferential allelic expression pattern in human skin. In infants, the paternal allele tends to express Dio3 more dominantly, while the maternal allele becomes more prominent in adults [37]. Most research on human lncRNA Dio3os centers on its altered expression in cancer cells, where it is typically downregulated, suggesting a potential tumor suppressor function across various cancers [16]. Recent studies have identified decreased Dio3os expression in hepatocellular carcinoma, thyroid carcinoma, and non-small cell lung cancer but abnormally increased in breast cancer [20] and pancreatic cancer [19]. Laura Magill Sack proposed that the structure of tissue-type-specific genetic networks underlies the selection of drivers in different cancers [38]. lncRNA Dio3os is differentially expressed in different cancers that may be related to tissue specificity. Cui *et al.* performed a meta-analysis of pancreatic cancer and non-tumour samples retrieved from the TCGA database. They measured the levels of Dio3os in pancreatic cancer cell lines and the normal pancreatic ductal epithelial cell line HPDE6-C7 [19]. The authors also investigated the association between Dio3os levels and overall survival in the TCGA PC dataset, and these data suggest that increased Dio3os expression may be associated with tumourigenesis or progression in pancreatic cancer [19]. Moreover, lncRNAs Dio3os, which are represented by lncRNAs, are influenced by epigenetic mechanisms. There is increasing evidence that epigenetics is susceptible to environmental changes that regulate individual growth, development, and disease by affecting chromatin activity and regulating gene expression [39]. Chen *et al.* found that long-term use of estrogen-deprivation drugs in a breast cancer study led to metabolic re-editing of breast cancer cells, which caused an up-regulation of Dio3os expression to promote cancer cell proliferation by increasing glycolysis [20]. Chen *et al.* also found that the increased expression of lncRNA Dio3os may be associated with pancreatic cancer tumour development or progression [20]. In ovariectomised Dio3os-overexpressing nude mice, Chen *et al.* administered estrogen and found that the tumour growth rate was significantly higher in the Dio3os group compared to the control group at low doses of estrogen [20].

Similarly, overexpression of Dio3os accelerated tumour growth when high-dose estrogen was administered [20]. In addition, overexpression of Dio3os significantly increased the tumourigenicity in nude mice treated with ovariectomy with complete estrogen deprivation and without estrogen tablets [20]. These results suggest that Dio3os positively regulates estrogen-independent breast cancer cell proliferation and glucose metabolism *in vivo*.

## 3 ROLE OF DIO3OS AND POTENTIAL MOLECULAR MECHANISMS IN GLUCOSE METABOLISM

### 3.1 Dio3os Promotes Glycolysis in Pancreatic Cancer Cells

**Fig. (1):**
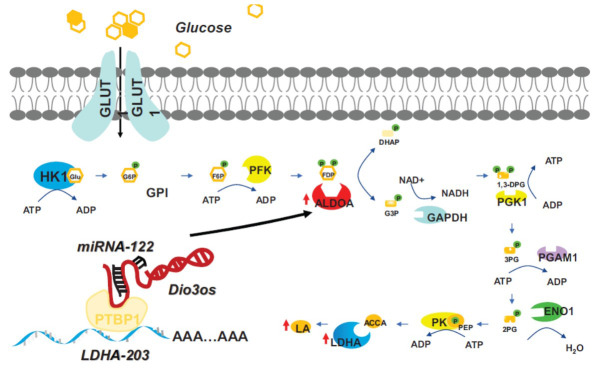
The Dio3os enhances glycolysis in cancer cells.

Aerobic glycolysis, a distinctive metabolic feature of cancer cells, is essential for maintaining cancer stem cell status and contributes to cancer progression, migration, and drug resistance [40]. Oncogenic mutations typically lead to the upregulation of glucose transporter proteins like GLUT1, which enhances glucose metabolism in cancer cells to support their malignant growth [41]. A 2019 study by Cui *et al.* found elevated lncRNA Dio3os levels in pancreatic cancer tissues and cell lines [19]. This study demonstrated that Dio3os competes with microRNA-122 (miR-122) for binding, thereby inhibiting its function and decreasing miR-122 levels [19]. The microRNA MiR-122, primarily abundant in the liver, holds a significant position in metabolic regulation. Song K *et al.* showed that miR-122 could regulate PDK4, thereby inhibiting glycolysis and lipid droplet formation in hepatocellular carcinoma, suggesting a potential therapeutic target [42]. The interplay between Dio3os and miR-122 seems to affect metabolic pathways. ALDOA, an enzyme crucial in glycolysis, is upregulated in various cancers [43]. Cui *et al.* identified that miR-122 is directly targeted by ALDOA [19]. Their findings suggest that Dio3os competes with miR-122 as an endogenous RNA, subsequently leading to increased expression levels of ALDOA [19]. Consequently, the Dio3os/miR-122/ALDOA pathway is a significant focus for exploring the regulation of glycolysis in cancer (Fig. **1**).

### 3.2 Dio3os Promotes Glycolysis Leading to Drug Resistance in Breast Cancer Cells

In 2022, Chen *et al.* identified a new mechanism contributing to drug resistance in breast cancer patients treated with aromatase inhibitors (AIs) [20]. Aromatase inhibitors block the enzyme aromatase, which converts androgens into estrogens, a key regulatory step. A significant proportion of breast cancer cases, approximately one-third, demonstrate the expression of estrogen receptor-alpha (ERα), which is responsible for driving tumor growth in the presence of estrogen [44]. AIs effectively lower estrogen levels, inhibiting the proliferation of estrogen receptor-positive (ER+) breast cancer cells [20]. The research showed Dio3os expression upregulation and enhanced aerobic glycolysis in long-term estrogen-deprived, drug-resistant breast cancer cells [20], underscoring the role of Dio3os in regulating cancer glycolysis. They unveiled an interaction between Dio3os and polypyrimidine tract-binding protein 1 (PTBP1) [20], a splicing suppressor that affects RNA processing and is linked to breast cancer cell proliferation and glycolysis [45, 46]. The study identified lactate dehydrogenase A (LDHA), crucial in the glycolysis pathway, as a downstream target affected by the Dio3os-PTBP1 interaction [47]. LDHA is essential in converting pyruvate to lactate, which not only supports cancer growth but also serves as a signaling molecule that promotes angiogenesis, invasion, migration, and immune evasion [47-49]. Knocking down Dio3os or PTBP1 led to a shift to unstable LDHA variants, emphasizing the Dio3os-PTBP1 interaction's role in maintaining LDHA mRNA stability through 3'UTR integrity and thereby enhancing glycolysis in drug-resistant cancer cells (Fig. **1**) [20].

### 3.3 Role of Dio3os in Diabetic Peripheral Neuropathy Development

Diabetic peripheral neuropathy (DPN) is a common complication among diabetes patients, marked by a range of physiological and pathological alterations, including nerve fiber loss, axonal degeneration, inflammation, cellular metabolism, redox balance disturbances, enhanced capillary permeability, limited nerve blood flow, microvascular damage, neurodegeneration, and compromised nerve signaling [50-53]. Yua *et al.* performed a biological analysis of BSK Sciatic Nerve (BSK SCN) Affymetrix microarray data from mice afflicted with DPN [23], leading to the re-identification of differentially expressed lncRNAs in DPN, with Dio3os being one of them [23]. Subsequent GO and KEGG analyses indicated a link between Dio3os and the Mitogen-Activated Protein Kinase (MAPK) cascade [23]. Earlier, Yu *et al.* observed a notable negative correlation between Dio3os and A530053G22RIK expressions in the KEGG pathway analysis [23]. Moreover, Chang YC *et al.* underscored the involvement of the MAPK/ERK/JNK/P38 pathway in DPN and related diabetic complications [43]. Wang *et al.* provided insights into the reduced expression of Dio3os under inflammatory conditions [54]. Wallace *et al.* previously established a significant connection between Dio3os and type I diabetes susceptibility [55]. In 2013, Velez Edwards *et al.* found that the SNPrs8008758 locus in Dio3os interacts with alcohol to influence body mass index [35]. Dongdong Yua *et al.* further discussed the potential influence of Dio3os and A530053G22Rik on DPN progression *via* the MAPK pathway [23].

## 4 ROLE OF DIO3OS AND POTENTIAL MOLECULAR MECHANISMS IN LIPID METABOLISM

### 4.1 The lncRNA Dio3os and Obesity

The rising prevalence of obesity alongside higher living standards has underscored a robust correlation between Dio3os and obesity. Hernandez *et al.* discovered in 2007 that Dio3os expression correlated with mature brown adipocytes in rats [56]. Additionally, Xie *et al.* demonstrated Dio3os's role in body weight regulation through studies on mouse endometrial stromal cells [57]. Recent research highlighted Dio3os expression suppression in the Brown Adipose Tissue (BAT) of offspring from obese mothers, which affects BAT development and contributes to obesity across generations [21].

### 4.2 Dio3os's Mediation of ZEB1 *Via* NONO Proteins in Obesity Control

**Fig. (2):**
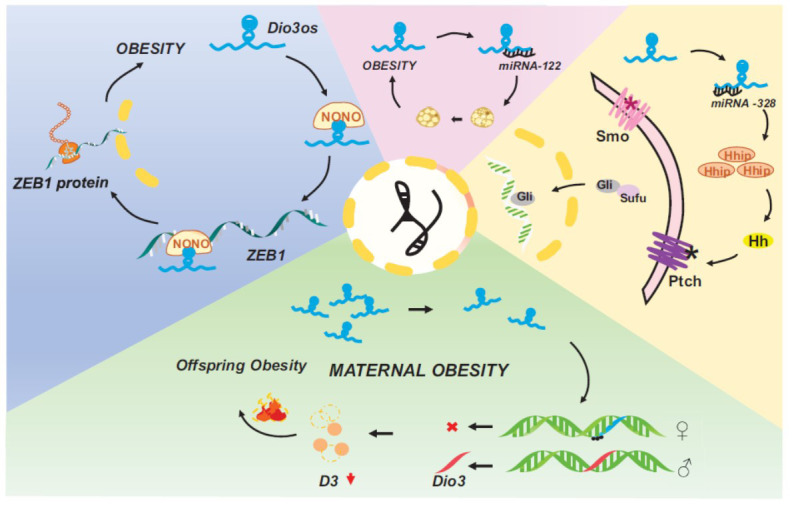
The role of long noncoding RNA Dio3os in obesity.

Hou *et al.* discovered that Dio3os significantly inhibits stemness in hepatocellular carcinoma (HCC) [16], noting its trans-regulatory effect on ZEB1, a transcription factor implicated in epithelial-mesenchymal transition (EMT) and tumor stemness [58]. The research revealed that Dio3os's regulation of ZEB1 entails the mediation of NONO, a protein predominantly facilitating mRNA nuclear retention within nuclear paraspeckles [16]. NONO proteins interact with ZEB1, facilitating the nuclear export of ZEB1 mRNA and impacting ZEB1 protein translation [16]. The findings of Hou *et al.* suggest that Dio3os suppresses the stemness of hepatocellular carcinoma cells by interacting with NONO proteins, thereby reducing the nuclear export of ZEB1 mRNA and diminishing the functionality of ZEB1 [16]. Given previous studies demonstrating the involvement of ZEB1 in obesity [59-61], the authors propose that Dio3os may contribute to obesity by trans-regulating ZEB1 (Fig. **2**) [16].

### 4.3 Impact of Maternal Obesity-induced Dio3os Suppression on Intergenerational Obesity

Stuebe AM *et al.* underscored the role of maternal obesity (MO) in the ongoing obesity epidemic, highlighting its correlation with obesity and metabolic dysfunction in offspring [62]. Yan-Ting Chen *et al.* demonstrated that maternal obesity adversely affects the development of fetal brown adipose tissue, resulting in reduced thermogenic capacity and metabolic disorders in female offspring [21]. Disruption of internal cellular communication for thyroid hormone in brown adipose tissue significantly impacts its growth and heat production [63]. Dio3, responsible for deiodinase 3 (D3) synthesis, is crucial for maintaining intracellular thyroxine balance [7]. The team observed a reduction in Dio3os expression, an imprinted gene influencing thyroid hormone metabolism, in the brown adipose tissue of offspring from obese mothers [21]. They identified DNA hypermethylation in the Dio3os promoter region within oocytes, fetuses, and offspring brown adipose tissue, persisting and resulting in Dio3os suppression and paternal Dio3 allele activation. This led to decreased D3 levels and attenuated brown adipose tissue thermogenesis [21, 49]. Consequently, these alterations predisposed female offspring to obesity and metabolic disorders (Fig. **2**).

### 4.4 Role of Dio3os in Regulating Lipid Metabolism Through MiR-122 Binding

Furthermore, Kang Cui *et al.* discovered that Dio3os is upregulated in pancreatic cancer cells and acts as a ceRNA with miR-122, forming a regulatory network that directly affects cell proliferation by interacting with miR-122 [19]. Considering the crucial role of miR-122 in regulating hepatic cholesterol and fatty acid metabolism, its interaction with Dio3os may influence lipid metabolism and obesity [64]. Yan-Ting Chen *et al.* suggested the necessity for further investigation to elucidate how the Dio3os-miR-122 interaction potentially modulates Dio3 expression and impacts lipid metabolism (Fig. **2**) [21].

### 4.5 Role of Dio3os/MiR-328/Hhip Axis in Regulating Hh Signaling Pathway During Adipocyte Differentiation

Zhanpeng Wang *et al.* observed diminished Dio3os expression in HCC tissues and cell lines. They demonstrated that Dio3os functions as a ceRNA by sequestering miR-328, consequently increasing Hhip expression [64, 65]. Zuo Y *et al.* identified Hhip as a negative regulator of the Hedgehog (Hh) pathway [66]. Furthermore, Wang *et al.* established the Dio3os/miR-328/Hhip axis as a modulator in HCC, wherein Dio3os-induced upregulation of Hhip disrupts the Hh pathway, leading to decreased levels of Gli1, Gli2, and Gli3 proteins [64, 65]. Wei H *et al.* noted low Hhip expression in adipose tissue during development, and the introduction of recombinant Hhip enhances adipocyte differentiation through the Hh signaling pathway, characterized by lipid accumulation and increased expression of GLUT4 and PPARγ [67]. Thus, the Dio3os/miR-328/Hhip pathway is proposed to influence adipocyte differentiation by regulating the Hh signaling pathway (Fig. **2**).

### 4.6 Atherosclerosis

Atherosclerosis (AS) encompasses a group of conditions characterized by the accumulation of metabolic substances, such as lipids and inflammatory secretions, within the inner walls of arterial blood vessels, resulting in vessel hardening and narrowing, thus impeding blood flow. lncRNAs play a significant role in cardiovascular disorders by influencing genes associated with endothelial dysfunction and smooth muscle function [67, 68], which are crucial for cell proliferation, macrophage activity, and lipid metabolism, all contributing to the initiation and progression of atherosclerotic disease (AD) [68]. Through their comparative analysis of gene expression between atherosclerotic plaques (CAP) and internal mammary artery tissue (IMA), Serdal Arslan *et al.* discovered a reduction in the expression of lncRNA Dio3os [22]. This finding suggests that Dio3os may have a regulatory function in the development of atherosclerotic plaques.

## 5 ROLE OF DIO3OS AND POTENTIAL MOLECULAR MECHANISMS IN OTHER CANCERS

Based on the genome-wide expression pattern of lncRNAs in different tissues and their tissue-specific expression characteristics, there is increasing evidence that altered lncRNA expression and its mutations are closely related to tumourigenesis and metastasis, energy metabolism, and drug resistance [69, 70]. The LncRNA Dio3os has recently been reported to play a regulatory role in a variety of cancers.

### 5.1 Dio3os Inhibits Hepatocellular Carcinoma Progression

Several literature reports have described the downregulation of LncRNA Dio3os in HCC tissues and cells and its inhibition of HCC progression through various mechanisms. Ya-Rui Hou *et al.* found that lncRNA Dio3os was downregulated in HCC and that it could bind to NONO protein and inhibit zinc finger box binding homology box 1 (ZEB1) from the nucleus, which significantly inhibited tumour progression through its inhibitory effect in HCC hepatocytes [16]. Zhanpeng Wang *et al.* showed that Dio3os could disrupt the HH pathway with miRNA 328-mediated HH-interacting protein (HHH), which inhibited hepatocellular carcinoma [65]. In 2023, Yunhan Wang *et al.* further found that the downregulation of lncRNA Dio3os in hepatocellular carcinoma cells was associated with their immune infiltration [71]. However, the glycolytic role of Dio3os in HCC has not been clarified. Other lncRNAs have been reported to be involved in HCC glycolysis by regulating key glycolytic enzymes and related transcription factors, such as lncRNA Ftx, which can inhibit the expression of pyruvate dehydrogenase kinase 1 (PDK1) to promote aerobic glycolysis in HCC [72]. lncRNA HOTAIR was found in HCC cells after hypoxia treatment [73]. The expression of lncRNA HOTAIR was downregulated in hypoxia-treated HCC cells and inhibited glycolysis by regulating the expression of miR-130a-3p, which in turn downregulated the expression of HIF-1α [73]. In 2023, the lncRNA HClnc1 was found to interact with PKM2 and prevent its degradation, thereby promoting PKM2-STAT3 signalling and glycolysis in HCC cells [72].

### 5.2 Dio3os and Thyroid Cancer

In 2022, Wang Y *et al.* confirmed that the study found that the expression of Dio3os was low in papillary thyroid carcinoma, and a positive correlation was found between Dio3os and immune cell infiltration, which may inhibit the progression of papillary thyroid carcinoma through the immune-inflammatory signalling pathway [74]. However, an article in 2020 suggested that Dio3os is elevated in thyroid cancer and, as an oncogenic factor in thyroid cancer, may regulate the expression of Nf-KYB2 through the Dio3o/Lt-7d axis, thereby affecting cell viability, DNA synthesis, infiltration, and migration of thyroid cancer cells [18]. Papillary thyroid carcinoma is a type of thyroid cancer, and the expression of LncRNA Dio3os in thyroid cancer is opposite to that in papillary thyroid carcinoma, and it is necessary to explore whether this differential expression is due to the differentiation of its cancer sites. At present, there is a lack of research into the glycolysis of Dio3os in thyroid cancer. Previous studies have found that lncRNA GLTC [75] and LNCRNA00671 [76] can affect lactate dehydrogenase A (LDHA)in different ways, thereby promoting LDHA enzyme activity. This increases glycolysis and proliferation of thyroid papillary carcinoma.

### 5.3 Dio3os Promotes Osteosarcoma Metastasis

Jinghong Yuan *et al.* found that LncRNA dio3os was upregulated in osteosarcoma [77]. After the combined use of Dio3os silencing and TGF-β signaling pathway activator (TGF-β1), the results revealed that acting the TGF-β signaling pathway restored the affection of Dio3os silencing on inhibiting the metastasis, migration, and invasion of osteosarcoma *in vivo* and *in vitro* [77]. Therefore, lncRNA Dio3os promotes osteosarcoma metastasis by activating the TGF-receptor signaling pathway. Although further studies are needed to determine whether Dio3os promotes osteosarcoma proliferation, existing studies indicate that lncRNAs, such as HAND2-AS1 [78], lncRNA SARCC [79] and lncRNA GAS5 [80], are involved in the glycolytic process in osteosarcoma.

### 5.4 Dio3os Inhibited Non-small Cell Lung Cancer Progression

In 2021, a study showed that Dio3os inhibited tumour growth in NSCLC by competitively binding to hnRNPK [17]. Hao-Shuai Yang *et al.* found that Dio3os has been validated by FISH experiments in tissues of driver-negative LUAD patients and found to be strongly associated with ESTIMATE, stromal/immune, and nucleic acid stemness scores [81]. There are not many studies on lncRNA dio3os in non-small cell lung cancer. As for other non-coding RNAs, LINC01123 [82] and lncRNA-AC020978 [83] have been reported to promote proliferation and aerobic glycolysis in NSCLC.

## 6 THE CERNA NETWORK REGULATORY MECHANISM OF DIO3OS

Recent research developments have increasingly emphasized the ceRNA network, representing a sophisticated regulatory system involving interactions among non-coding RNAs and coding RNAs through miRNAs. This network provides a novel perspective and investigative avenue for comprehending the mechanisms underlying various diseases. The core concept of ceRNA entails lncRNAs competing for miRNA binding, thereby modulating mRNA expression across diverse disease contexts (a novel form of RNA interplay) [84]. Furthermore, a single miRNA has the ability to regulate multiple mRNAs and lncRNAs, thereby playing a pivotal role in constructing a complex regulatory network. This network enables cells to meticulously modulate gene expression and adjust to diverse physiological states.

### 6.1 LncRNA-miRNA CeRNA Regulatory Network

The lncRNA Dio3os is implicated in regulating miR-4750-5p, which subsequently may indirectly affect the expression of the COL9A3 gene through a sponge-like mechanism. COL9A3 encodes a component of collagen IX, which is linked to the development of ovarian and uterine atopic disease [28]. Additionally, Dio3os's interaction with microRNA-130b facilitates the upregulation of PAX9, thereby restoring g radiosensitivity in esophageal squamous cell carcinoma [85]. In ovarian cancer, a competitive signaling pathway involving serum exosome Dio3os, miR-27a-3p, and HOXA10 influences transcriptional pathways, impacting disease progression and patient survival [86]. Dio3os impedes the proliferation and spread of hepatocellular carcinoma cells by competitively interacting with miR-328, leading to the downregulation of Hhip [52]. Blocking the Dio3os/let-7d/NF-κB2 pathway results in suppressed Dio3os levels, which in turn decreases the expressions of ki-67 and PCNA, ultimately leading to a reduction in cancer cell viability [77]. Activation of the TGF-β signaling pathway serves to antagonize the silencing of Dio3os, thus effectively suppressing the metastatic, migratory, and invasive properties of osteosarcoma [77]. The Dio3os/let-7d/NF-κB2 axis also plays a crucial role in thyroid cancer cell proliferation and metastasis [18]. Dio3os is significantly upregulated in pancreatic cancer, playing a pivotal role in regulating cell proliferation and metastasis through the miR-122/ALDOA axis [37]. It also modulates the expression of CTGF and ZEB1 *via* miR-656-3p and miR-485-5p, promoting BPH-1 cell proliferation and inducing epithelial-mesenchymal transition in WPMY-1 cells [87]. Furthermore, Dio3os serves as a chromatin localizer, impeding the interaction between proteins and DNA regions, specifically targeting miR-18a-3p, miR-1913, and miR-266-3p [88].

### 6.2 LncRNA-mRNA Regulatory Network

The lncRNA Dio3os exerts a downregulatory effect on the expression of MSX2, BEND3, and SH2D3A, highlighting its involvement in the progression of ovarian endometriosis [28].

### 6.3 Other Mechanisms

Dio3os significantly reduces the expression of MYC and its downstream target, CDC25A, by competitively binding to heterogeneous ribonucleoprotein K (hnRNPK) [17]. This binding impedes the interaction of hnRNPK with MYC DNA and MYC mRNA, consequently inhibiting MYC transcription and translation, thereby playing a role in the progression of lung small cell carcinoma (NSCLC) *via* the hnRNPK-MYC-CDC25A pathway [17]. Additionally, Dio3os is targeted by EZH2 in cancer, resulting in the suppression of Dio3os expression [30]. A recent study emphasized the interaction between Dio3os and the NONO protein, which impedes the NONO-mediated nuclear export of ZEB1 mRNA, a crucial pathway in attenuating hepatocellular carcinoma progression [47]. Furthermore, Dio3os interacts with polypyrimidine tract binding protein 1 (PTBP1) to facilitate a metabolic shift towards glycolysis in breast cancer cells, thereby promoting increased expression of lactate dehydrogenase A (LDHA) [20].

## 7 DISCUSSION

As transcriptional studies progress, a class of non-coding RNAs has emerged as a significant regulator in gene expression, with lncRNAs playing a crucial role in maintaining physiological structure, growth, development, and various cellular functions. The regulatory activities of lncRNAs are key factors that significantly impact the development of various diseases, including immunological conditions, cancers, and inflammatory responses. This important function positions them as promising candidates for therapeutic research and interventions. Notably, the exploration of lncRNAs in metabolism remains relatively understudied. This paper focuses specifically on elucidating the current understanding of lncRNA Dio3os in metabolism, offering a fresh perspective for lncRNA research in this domain. Existing studies suggest that lncRNA Dio3os serves not only as a potential biomarker for cancer but also plays a significant role in regulating metabolic functions, opening new avenues for clinical therapeutic target exploration.

A significant aspect of lncRNA research is its specificity. Unlike traditional mRNA studies, lncRNAs demonstrate tissue-specific and cell-specific characteristics [89]. For instance, a 2017 CRISPR study identified 499 lncRNAs crucial for cell growth, with 89% functioning uniquely within specific cell types [90]. Knocking down lncRNA expression can distinctly influence gene expression and functionality in targeted cell types [90]. Olivia M. DeGoede *et al.* compared lncRNAs across various tissues, identifying 316 lncRNAs exclusively expressed in a single tissue, with the highest abundance found in the testis, brain, blood, and skin [91]. This unique expression profile is vital for maintaining tissue structure and managing tissue-specific disease progression. Tissue-specific lncRNAs could serve as biomarkers to distinguish between tissues, and their particularity affects tissue-related pathways, significantly influencing disease research [92]. For instance, the upregulation of lncRNA FOXd2-AS1 in colorectal cancer tissues suggests a strong link between this elevation and tumor cell differentiation, highlighting the potential of lncRNA FOXd2-AS1 as a diagnostic marker in colorectal cancer [93]. This study provides an initial analysis of lncRNA Dio3os's expression pattern, noting that Dio3os, an imprinted gene, exhibits co-expression traits. However, the specific mechanisms and phenotypes regulated by its expression lack robust experimental support and require further exploration.

An in-depth examination of lncRNAs elucidates their complex roles in gene expression regulation and cellular functions, particularly through the formation of ceRNA regulatory networks, shedding light on the molecular mechanisms that govern physiological and disease processes and offering valuable insights for clinical phenotype, pharmacology, and pathology research. This study reviews the identified lncRNA Dio3os-related ceRNA pathways, noting a predominant focus on cancer phenotypes and a scarcity of robust evidence for the establishment of metabolism-associated ceRNA networks. Additionally, the analysis of glycolipid metabolism-related pathways presented here introduces fresh perspectives for future investigations.

In the expanding field of genetic research, the potential for nucleic acid-targeted therapeutics involving lncRNAs is increasingly recognized [94]. Due to their unique physiological properties, lncRNAs can interact with DNA, RNA, and proteins, enabling multi-targeted drug actions. The tissue-specific and cell-specific expression patterns of lncRNAs minimize the side effects typically associated with conventional drugs, highlighting their significant potential in novel drug development. Currently, only resveratrol [95] and zearalenone [24] have been reported to regulate lncRNA Dio3os, indicating that the development of Dio3os-targeted drugs is still in its infancy.

## CONCLUSION

In conclusion, the participation of lncRNA Dio3os in glycolipid metabolism enhances our comprehension of this biological process and its genetic interrelations, paving the way for novel approaches in the development of lncRNA-based therapeutic strategies.

## AUTHORS’ CONTRIBUTIONS

All authors contributed to the writing of this review. The first draft of the manuscript was written by Xinchen Wang, and all authors commented on previous versions of the manuscript. All authors read and approved the final manuscript.

## Figures and Tables

**Table 1:** The regulatory functions of Dio3os in glycolipid metabolic diseases.

**References**	**Biological Function**	**Type of Diseases**	**Expression**	**Related Gene/** **protein**	**Downstream Target**	**Signaling Pathways**
Cui *et al*., 2019 [19]	Cancer glycolysis	Pancreatic cancer cells	Up	MicroRNA-122	ALDOA	Dio3os/miR-122/ ALDOA
Chen *et al*., 2022 [20]	Enhancing glycolysis in drug-resistant cancer cell	Breast cancer cells	Up	PTBP1	LDHA	Dio3os/PTBP1/LDHA
Yu *et al*., 2023 [23]	Development of diabetic peripheral neuropathy	Diabetic peripheral neuropathy	Down	A530053G22Rik	MAPK	Dio3os/A530053G22Rik/MAPK
Hou *et al*., 2023 [16]	Inhibition of cancer cell stemness, Obesity	Hepatocellular carcinoma	Down	NONO	ZEB1	Dio3os/NONO/ZEB1
Chen *et al*., 2021 [21]	Intergenerational obesity	Maternal obesity	Down	Dio3	Deiodinase 3	Dio3os/Dio3/ Deiodinase 3
Cui *et al*., 2019 [19] Chen *et al*., 2021 [21]	Lipid metabolism	Pancreatic cancer cells	Up	MicroRNA-122	Dio3	Dio3os/miR-122/Dio3
Wang *et al*., 2020 [65]	Hinderscell malignant behaviors, Adipocyte differentiation	Hepatocellular carcinoma	Down	MicroRNA-328	Hhip, Hh signaling pathway	Dio3os/miR-328/Hhip axis regulates Hh signaling pathway
Arslan *et al*., 2023 [22]	Atherosclerotic plaque formation	Atherosclerosis	Down	-	-	-

## LIST OF ABBREVIATIONS

1,3-DPG: 1,3-Bisphoshoglycerate
2PG: 2-Posphoglycerate
3PG: 3-Posphoglycerate
ACCA: Pyruvate
AD: Atherosclerotic Disease
AIs: Aromatase Inhibitors
ALDOA: Aldolase A
AS: Atherosclerosis
BAT: Brown Adipose Tissue
BRCA: Breast Cancer
CAP: Atherosclerotic Plaques
ceRNA: Competing Endogenous RNA
COL9A3: Collagen, Type IX, Alpha 3
CTGF: Connective Tissue Growth Factor
D3: Deiodinase 3
DHAP: Dihydroxyacetone Phosphate
DPN: Diabetic Peripheral Neuropathy
EMT: Epithelial-mesenchymal Transition
ENO1: Enolase1
ER+: Estrogen Receptor-positive
ERα: Estrogen Receptor-alpha
EZH2: Enhancer of Zeste Homolog 2
F6P: Fructose-6-phosphate
FDP: Fructose-1,6-bisphosphate
G3P: Glyceraldehyde-3-phosphate
G6P: Glucose-6-phosphate
GAPDH: Glyceraldehyde 3-phosphatedehydrogenase
GBM: Glioblastoma Multiforme
Glu: Glucose
GPI: Glucose-6-phosphate Isomerase
H3K27: Histone H3's lysine 27
HCC: Hepatocellular Carcinoma
Hh: Hedgehog
HK1: Hexokinase
hnRNPK: Heterogeneous Ribonucleoprotein K
HOXA10: Homeobox A10
IMA: Internal Mammary Artery Tissue
LA: Lactic Acid
LDHA: Lactate Dehydrogenase A
LncRNA: Long Non-coding RNA
MAPK: Mitogen-Activated Protein Kinase
miR-122: MicroRNA-122
MO: Maternal Obesity
NSCLC: Lung Small Cell Carcinoma
PAX9: Paired Box Gene 9
PCNA: Proliferating Cell Nuclear Antigen
PEP: Phosphoenolpyruvate
PFK: Phosphofructokinase
PGAM1: Phosphoglycerate Mutase1
PGK1: Phosphaglycerate Kinase1
PK: Pyruvate Kinase
PRAD: Prostate Cancer
PRC2: Polycomb Repression Complex 2
PTBP1: Polypyrimidine Tract-binding Protein 1
SCN: BSK Sciatic Nerve
TGF-β: Transforming Growth Factor Beta
ZEB1: Zinc Finger E-Box Binding Homeobox 1
